# Credit card fraud detection using a hierarchical behavior-knowledge space model

**DOI:** 10.1371/journal.pone.0260579

**Published:** 2022-01-20

**Authors:** Asoke K. Nandi, Kuldeep Kaur Randhawa, Hong Siang Chua, Manjeevan Seera, Chee Peng Lim

**Affiliations:** 1 Department of Electronic and Electrical Engineering, Brunel University London, Uxbridge, UB8 3PH, United Kingdom; 2 Visiting Professor, School of Electronic and Information Engineering, Tongji University, Shanghai, China; 3 Faculty of Engineering, Computing and Science, Swinburne University of Technology (Sarawak Campus), Malaysia; 4 Econometrics and Business Statistics, School of Business, Monash University Malaysia, Selangor, Malaysia; 5 Institute for Intelligent Systems Research and Innovation, Deakin University, Geelong, Victoria, Australia; Universitat Politecnica de Catalunya, SPAIN

## Abstract

With the advancement in machine learning, researchers continue to devise and implement effective intelligent methods for fraud detection in the financial sector. Indeed, credit card fraud leads to billions of dollars in losses for merchants every year. In this paper, a multi-classifier framework is designed to address the challenges of credit card fraud detections. An ensemble model with multiple machine learning classification algorithms is designed, in which the Behavior-Knowledge Space (BKS) is leveraged to combine the predictions from multiple classifiers. To ascertain the effectiveness of the developed ensemble model, publicly available data sets as well as real financial records are employed for performance evaluations. Through statistical tests, the results positively indicate the effectiveness of the developed model as compared with the commonly used majority voting method for combination of predictions from multiple classifiers in tackling noisy data classification as well as credit card fraud detection problems.

## 1. Introduction

Classification has been a key application area of machine learning. A classifier learns a mathematical model from training data samples that maps input features to the target classes or labels [1]. Given a new unseen data sample, the trained classifier is used to provide a prediction of the target class [2]. It is, however, not easy to use single or few input variables only to differentiate multiple classes to their fullest [1]. In many classifiers such as neural networks, *k*-nearest neighbors (*k*NN), Support Vector Machine (SVM), and Naïve Bayes (NB), the underlying assumption is that training data samples contain a valid representation of the population of interest, which normally require a balanced sample distribution [3]. It has been empirically observed that building an accurate classifier based on a single paradigm is often ineffective, if not impossible [2].

Establishing an accurate classifier is not an easy task, as each classification method has its own advantages and disadvantages. As a result, the concept of classifier fusion using multiple classifiers has become one of the most significant methodologies to improve the classification performance. All classifiers provide their predictions of the class of an incoming data sample, and these predictions are analyzed and combined using some fusion strategy [4]. In this regard, selections of appropriate classifiers for constructing an ensemble classification model remain a difficult task [2].

It is a well-established notion in the literature that a classifier combination offers a viable alternative to yield better results than those from a single classifier. This is however dependent on how independent and diverse the classifiers are. Diversity among the chosen classifiers is an important factor for building a successful multi-classifier system (MCS). Various MCS methods have been proposed in modelling and handling different types of data [5]. Research in this area has led to the development of MCS models that combine the strengths of various individual classifiers, which are built using different training paradigms, to provide improved and robust classification performance [2].

With the rapid growth in e-commerce, the number of credit card transactions has been on the rise [6]. Alongside this growth, the issue of credit card fraud has become serious and complicated [7]. Generally, fraud detection solutions can be divided into supervised and unsupervised classification methods [8]. In supervised methods, the classification models are based on different samples of genuine and fraudulent transactions, while in unsupervised methods, outliers are detected from the data samples [9]. Merchants are responsible for paying the bill when a fraud occurs through an online or in-store transaction [10]. In this paper, we focus on the design and application of an ensemble classification model for credit card fraud detection, which is regarded as a significant problem in the financial sector. Indeed, billions of dollars are lost annually due to credit card fraud, and both merchants and consumers are significantly affected by the consequences of fraud [11]. With the advancement in fraud detection methods, fraudsters are finding new methods to avoid detection. Capturing irregular transaction patterns is a vital step in fraud detection [12], and efficient and effective classification methods are required for accurate detection of credit card frauds.

Two main methods are compared, namely majority voting and Behavior-Knowledge Space (BKS) [13] in this paper. Majority voting is simple but effective method, where an odd number of constituent classifiers is used for a decision in an ensemble. On the other hand, BKS considers the predictive accuracy of each classifiers and use this extra information to aggregate predictions from individual classifiers and derive better results. The main contribution of this paper is the formulation of an ensemble MCS model with the BKS for detection of real-world credit card fraud. The proposed model allows the MCS to accumulate its knowledge and yield better results over time.

The organization of this paper is as follows. A literature review on different types of MCS is presented in Section 2. Designs of the MCS model with BKS are explained in Section 3. A series of empirical evaluation on credit card fraud using publicly available data as well as real-world data from our collection is presented in Section 4. A summary of the findings is given in Section 5.

## 2. Literature review

An MCS model commonly includes a decision combination method for combining predictions from an ensemble of classifiers. A number of applications using MCS models have been developed over the years. In this section, we present a literature review on different classifier configurations, starting from two classifiers to four or more classifiers.

### 2.1 Two classifiers

An ensemble classification model using *k*NN and SVM was presented in [14] to classify electrocardiogram (ECG) signals. The proposed model achieved an accuracy score of 0.752 as compared with 0.561 to 0.737 from other classifiers [14]. In financial market trading, an automated framework was presented in [15], and an MCS model was used a weighted multi-category generalized eigenvalue SVM and Random Forest (RF) to generate the buy or sell signals. Evaluated with five index returns, including those from NASDAQ and DOW JONES, the MCS model achieved notable improvements over the buy/hold strategy as compared with the outcomes from other algorithms [15].

Predictions of severity pertaining to abnormal aviation events with risk levels were conducted in [16] using an MCS framework consisting of SVM and deep learning models. The SVM was used for discovering the relationships between event synopsis and consequences, while deep learning was deployed in training. Using cross-validation, the proposed MCS model achieved 81% accuracy, which are 3% and 6% higher than standalone SVM and deep learning models, respectively [16].

In [17], an MCS model based on dynamic weights was developed. The MCS model comprised a backpropagation neural network and the nearest neighbour algorithm, which dynamically assigned a fusion weight to a classifier. Using several public face databases, the proposed method obtained better classification accuracy rates as compared with those from individual classifiers [17]. An MCS model was proposed for face image segmentation in [18]. A total of three Bayes and one SVM were used in the MCS model. An error rate of 13.9% was achieved, as compared with 50% from standard classifiers, for hair across eyes requirements [18].

### 2.2 Three classifiers

In [2], an MCS was designed using stacked generalization based on DT (Decision Tree), *k*NN, and NB. A total of 20 different UCI data sets were used in the experiments. Based on a breast cancer data set, an accuracy rate of 74.8% was achieved by the MCS model, as compared with 71.2% from other classifiers [2]. An adaptive MCS model for gene expression was examined in [4]. Particle swarm optimization, bat-inspired algorithm, and SVM were used in the ensemble model, which showed significant improvements in classification performance with respect to breast cancer and embryonal tumors, where the training error reduced by up to 50% [4].

In [19], an MCS model to maximize the diagnostic accuracy of thyroid detection. The model utilized SVM, NB, *k*NN, and closest matching rule classifiers to yield the best diagnostic accuracy. The proposed system achieved an accuracy of 99.5% as compared with 99.1% from the best individual classifier in automatically discriminating thyroid histopathology images as either normal thyroid or papillary thyroid carcinoma [19]. An MCS framework to exploit unlabelled data was detailed in [20]. The MCS model was built using NB, SVM, and *k*NN. A total of five text classification data sets were used in the experiments. The highest accuracy rate of 83.3% was achieved by the MCS model, as compared with those from other algorithms [20].

### 2.3 Four or more classifiers

An adaptive MCS model for oil-bearing reservoir recognition was presented in [5]. A total of five classifiers were used, namely C4.5, SVM, radial basis function, data gravitation-based, and *k*NN algorithms. A number of rules were included in the adaptive MCS model as well. The proposed solution achieved perfect accuracy in recognizing the properties of different layers in the oil logging data [5]. An advanced warning system was designed in [21] using an MCS approach for outward foreign direct investment. Logistic regression, SVM, NN, and decision trees were used in the MCS model, which was applied to resource-based enterprises in China. The experimental results indicated the MCS model was able to yield an accuracy score of 85.1%, as compared with 82.5% from a standard neural network model [21].

In [22], estimations of precipitation from satellite images were carried out with an MCS model, which combined RF, NN, SVM, NB, weighted *k*NN, and *k*-means together. A total of six classes of precipitation intensities were obtained, from no rain to very high precipitation. A score of 0.93 for the coefficient of correlation was yielded by the proposed method, as compared with only 0.46 from other methods [22]. In [23], a one-against-one method was explored using MCS that consisted of NN, DT, *k*NN, SVM, linear discriminant analysis, and logistic regression. An error rate of 0.99% was produced by the MCS model, as compared with 14.9% from other methods on the zoo data set [23]. In [24], sentiments of tweets are automatically classified either positive or negative using an ensemble. Public tweet sentiment datasets are used in the experiment. The ensemble is formed using multinomial NB, SVM, RF, and logistic regression. An accuracy rate of 81.06% was achieved on a dataset trained with only 0.03% of the obtained data [24].

### 2.5 Remarks

Based on the above review that focuses on various classifier configurations (from two or more classifiers), it is clear that MCS has been used in various applications, including finance, medical, engineering and other sectors. The MCS configuration offers the advantage that the output is not constrained by one classifier, with a pool of classifiers to provide the possibility of improved results. In the event that one classifier produces an incorrect prediction while other counterparts yield a correct one, the combined output can be correct, e.g. in accordance with the majority voting principle. The combined output is, therefore, able to reduce the number of incorrect predictions from single classification method. The results from various MCS configurations reported in the literature are promising, with typically higher accuracy rates. However, MCS-based methods tend to run slower, since a higher computation load is required for execution of multiple classifiers, although this is not regularly reported in the literature. While better results often outweigh longer computational durations, it is useful to ensure that MCS configurations are feasible in terms of computational requirements for practical applications in real-world environments.

## 3. Classification methods

In this study, several standard machine learning models from H2O.ai were employed to establish an MCS model. The Python software running on the Google Colab environment was used. In the following sub-sections, the majority voting and the BKS model by Huang and Suen [25] for decision combination is explained.

### 3.1 Majority voting

Given ***M*** target classes in which each class is represented by ***C***_***i***_, ∀_***i***_∈**Λ** = {**1**, **2**,…,***M***}. The classifier task is to categorize an input sample, ***x***, to one of the (***M***+**1**) classes, with the (***M***+**1**)th class denoting that the classifier rejects ***x***.

A commonly used method for combining multiple classifier outputs is by majority voting. If there are ***K*** classifiers, denoted by ***e***_**1**_,…,***e***_***K***_, the task is to produce a combined result, ***E***(***x***) = ***j*,*j***∈{**1**, **2**,…,***M***, ***M***+**1**} from all ***K*** predictions, ***e***_***k***_(***x***) = ***j***_***k***_, ***k*** = **1**,…,***K***. The number of votes can be computed using a binary function [26], i.e.,

$$V_{k}(x\in C_{i})=\left\{ \begin{array}{l} 1,\;if\;e_{k}(x)=i,i\in \Lambda \\ 0,\;otherwise. \end{array}\right.$$ (1)

Then, sum the votes from all ***K*** classifiers for each ***C***_***i***_

$$V_{K}(x\in C_{i})=\sum_{k=1}^{K}V_{k}(x\in C_{i}),\;i=1,\ldots ,M$$ (2)

and the combined result, ***E***(***x***), can be determined by

$$E(x)=\left\{ \begin{array}{l} j,\;if\;V_{E}(x\in C_{j})={max}_{i\in \Lambda }(x\in C_{i})\;and\;\frac{V_{E}(x\in C_{j})}{K}\geq \lambda \\ M+1,\;otherwise \end{array}\right.$$ (3)

where **0**≤***λ***≤**1** is a user-defined threshold that controls the confidence in the final decision [27].

### 3.2 BKS

A BKS is a ***K***-dimensional space, where every dimension indicates the decision (i.e., predicted class) from one classifier. The intersection of the decisions from ***K*** different classifiers occupies one unit in the BKS, e.g., **BKS**(***e***_**1**_(***x***) = ***j***_**1**_,…,***e***_***K***_(***x***) = ***j***_***K***_) denotes a unit where each ***e***_***k***_ produces a prediction ***j***_***k***_, ***k*** = **1**,…,***K***. In each BKS unit, there are ***M*** partitions (cells), which accumulate the number of data samples actually belonging to ***C***_***i***_.

Consider an example with two classifiers. A two-dimensional (2–D) BKS can be formed, as given in Table 1.

**Table 1:** Two-dimensional BKS.

*e*_1_	1	2	…	*M*+1
*e*_2_				
1	***U***_**11**_	***U***_**12**_	**…**	***U***_**1**(***M***+**1**)_
2	***U***_**21**_	***U***_**22**_	**…**	***U***_**2**(***M***+**1**)_
**⋮**	**⋮**	**⋮**	**⋱**	**⋮**
***M*+1**	***U***_(***M***+**1**)**1**_	***U***_(***M***+**1**)**2**_	**…**	***U***_(***M***+**1**)(***M***+**1)**_

Every BKS unit, ***U***_***ij***_, contains ***M*** cells, i.e., $n_{1}^{H},\ldots ,n_{M}^{H}$, where ***H*** represents the overall prediction ***e***_**1**_(***x***) = ***j***_**1**_,…,***e***_***K***_(***x***) = ***j***_***K***_. The total number of data samples belonging to each class is recorded in each $n_{1}^{H}$, ***i*** = **1**,…,***M***. When an input sample, ***x***, is shown, one of the BKS units is activated (also known as the focal unit) after obtaining the decisions from all ***K*** classifiers. As an example, ***U***_**34**_ becomes active as the focal unit if ***e***_**1**_(***x***) = **3** and ***e***_**2**_(***x***) = **4**. The total number of samples in the focal unit can be obtained by using

$$T(H)=\sum_{i=1}^{M}n_{i}^{H}$$ (4)

and the one with the highest number of samples is identified

$$R(H)=j,\;where\;n_{j}^{H}={max}_{i\in \Lambda }(n_{i}^{H})$$ (5)

The decision rule for determining the final outcome is

$$E(x)=\left\{ \begin{array}{l} R(H),\;if\;T(H)>0\;and\;\frac{n_{R(H)}^{H}}{T(H)}\geq \lambda \\ M+1,\;otherwise \end{array}\right.$$ (6)

where **0**≤***λ***≤**1** is a user-defined confidence threshold.

The BKS has similarity with the confusion matrix. With the Bayesian approach, multiplication of evidence from the confusion matrices is required to estimate the joint probability of ***K*** events when combining the predictions. This step is eliminated in the BKS method, where a final decision is reached by giving the input sample directly to the class that has gathered the greatest number of samples. This simple method of BKS gives a fast and efficient method for combining various decisions, as shown in [25] for classification of unconstrained handwritten numerals.

**Fig 1:**
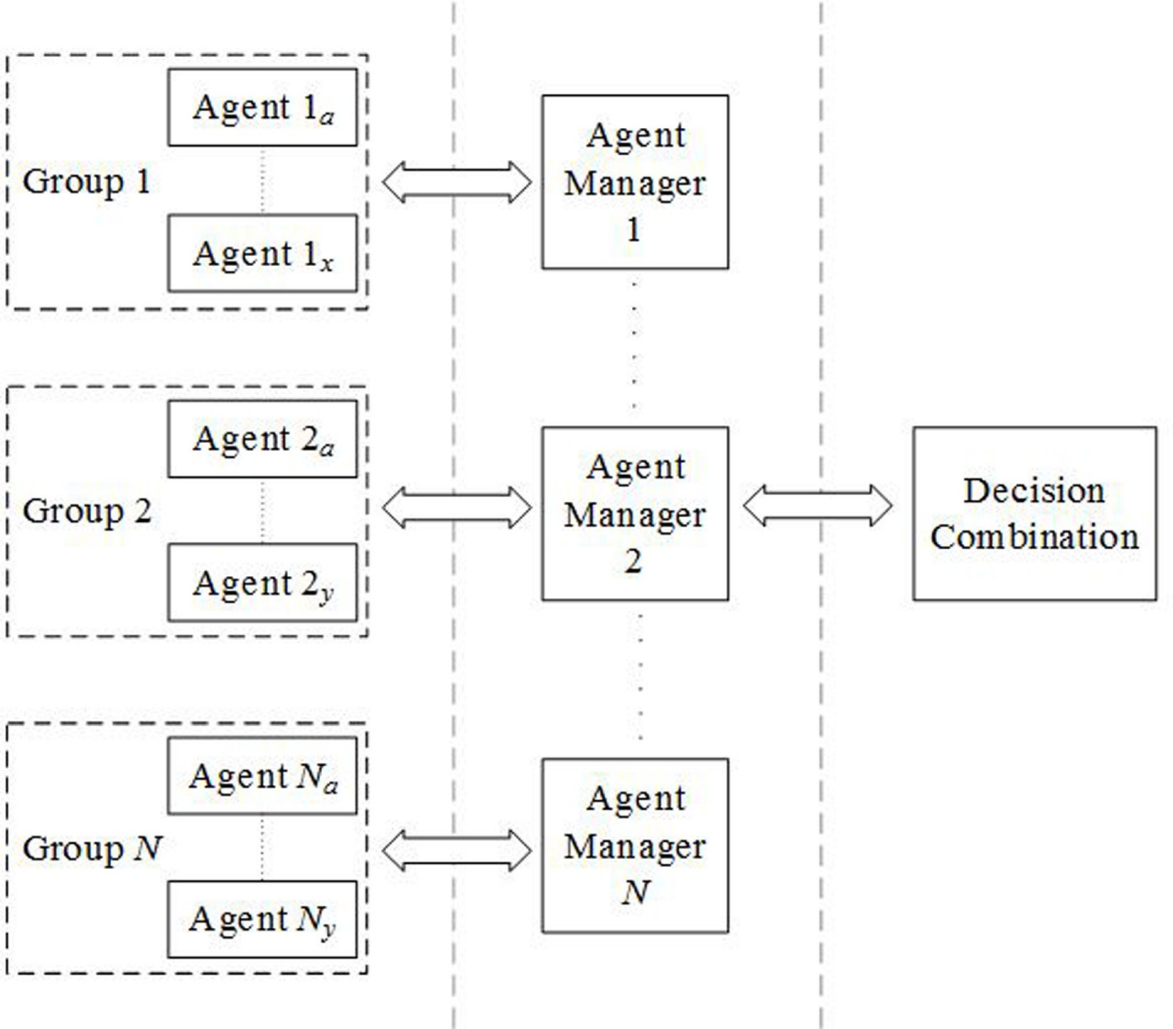
A hierarchical agent-based framework with the BKS.

A hierarchical agent-based framework with the BKS for decision combination is proposed. As shown in Fig 1, the framework has *N* agent groups in the base layer, with each group comprises multiple individual agents. The agents can be machine learning models, statistical methods as well as other classification algorithms. A manager agent is assigned to combine the predictions from each agent group using a BSK. Each manager agent sends its prediction to a decision combination module comprising another BKS in the top layer that produces the final combined prediction.

A numerical example is presented to better illustrate the BKS mechanism. In Table 2, a simple binary classification problem is shown. There are two agents (classifiers) and six input samples, along with their predicted and actual classes. A BKS can be constructed, as shown in Table 3. As an example, for input samples 1 and 4 (Table 2), both agents 1 and 2 predict class 1, and the actual class is 1. This information is recorded in the highlighted (grey) BKS unit in Table 3. Given a new test sample, the predictions from all agents are used to activate a BKS unit, and the combined predicted class (final output) is reached based on the highest number of samples from the majority class, as given in Eq (5). Whenever the highlighted (grey) BKS unit is activated during the test phase, the combined (final) prediction is Class 1.

**Table 2:** Prediction outputs of Agents 1 and 2.

Data	Actual class	Predicted Class	
		Agent 1	Agent 2
Sample 1	1	1	1
Sample 2	1	2	1
Sample 3	2	2	2
Sample 4	1	1	1
Sample 5	2	2	2
Sample 6	2	1	2

**Table 3:** Creation of BKS for the classification scenario in Table 2.

Agent 1	Predicted Class = 1	Predicted Class = 2
Agent 2		
Predicted Class = 1	No. of actual Class 1 samples = 2	No. of actual Class 1 samples = 1
	No. of actual Class 2 samples = 0	No. of actual Class 2 samples = 0
Predicted Class = 2	No. of actual Class 1 samples = 0	No. of actual Class 1 samples = 0
	No. of actual Class 2 samples = 1	No. of actual Class 2 samples = 2

## 4. Experiments

In this empirical evaluation, publicly available data sets from UCI Machine Learning Repository [28], KEEL Repository [29], and Kaggle [30] are used. A real-world data set is also used for evaluation.

### 4.1 Setup

**Fig 2:**
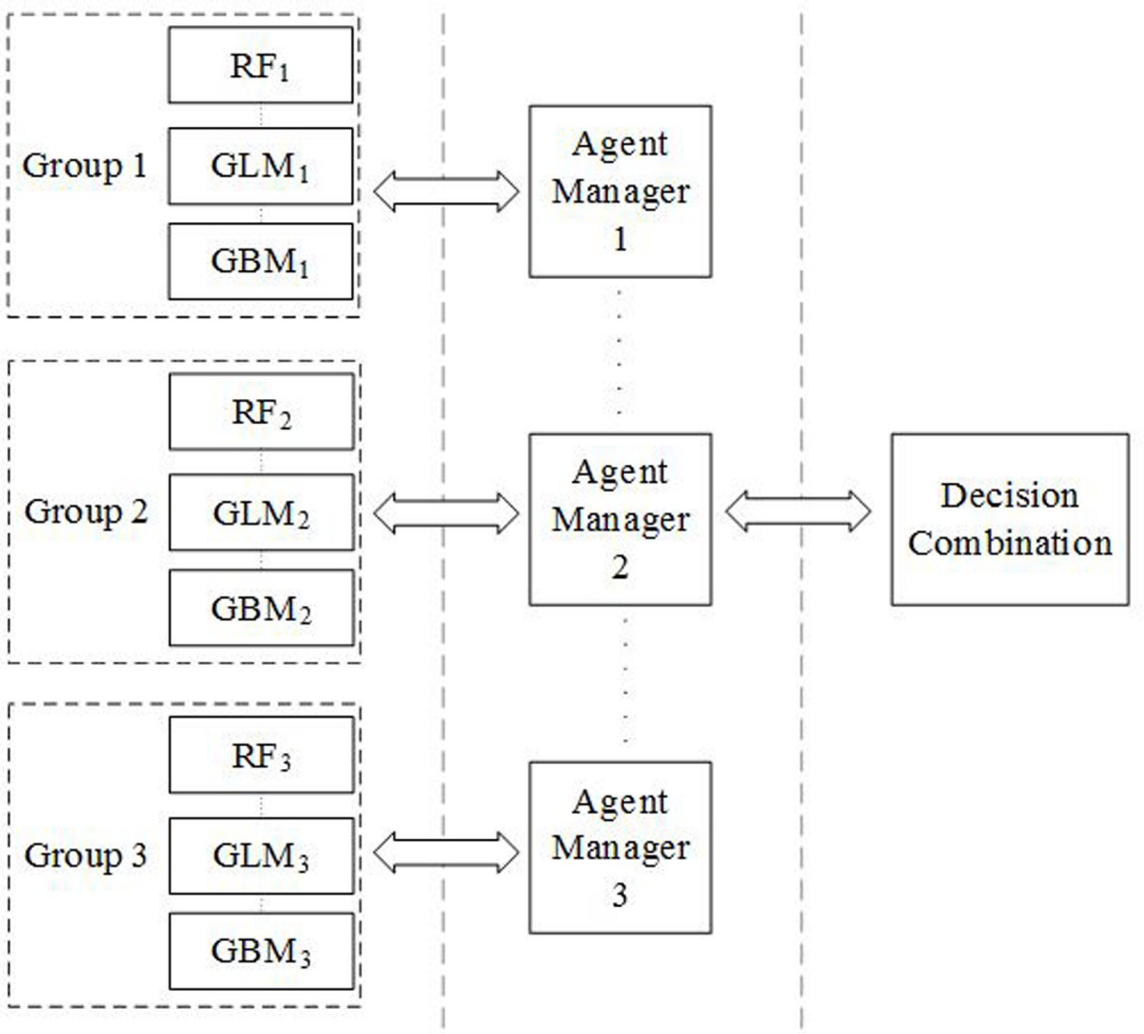
Configuration of the hierarchical agent-based framework used in the experiments.

Fig 2 shows the configuration of the hierarchical agent-based framework used in the experiments. It consists of three groups, where each group contains three agents. The three agents are Random Forest (RF), Generalized Linear Model (GLM), and Gradient Boosting Machine (GBM), which have been selected based on extensive experiments of individual and group performances. Three agent managers are established, each with a BKS module. The prediction from these three agent managers are sent to the decision combination module that has another BKS to produce the final predicted class.

Training is first conducted using randomized orders of the data samples, which is followed by a validation process. This in turn creates three group-based BKS modules (one for each group). The next step is combining the outputs from BKS modules 1 to 3 using training data with another randomized sequence, leading to the establishment of another overall (final) BKS module that combines the outputs from the previous three group-based BKS modules. Given a test sample, the group-based BKS outputs are combined again with the overall BKS module to produce a final predicted class for computation of the performance metrics, namely classification accuracy and F1-score.

Classification accuracy and F1-score of each experiment are recorded using Eqs (7) and (8), respectively.


$$Accuracy=\frac{TN+TP}{TP+FP+TN+FN}$$ (7)

$$F1=\frac{TP}{TP+\frac{1}{2}(FP+FN)}$$ (8)

For performance comparison between majority voting and BKS statistically, the sign test [31] is adopted. In the sign test, the number of wins is spread based on a binomial distribution. Given a large number of cases, the number of wins under the null hypothesis is distributed according to $n(\frac{n}{2},\frac{\sqrt{n}}{2})$, allowing the use of the *z*-test, i.e., should the number of wins be at least $\left(\frac{n}{2}+1.96\frac{\sqrt{n}}{2}\right)$, then the outcome is statistically significant with *p* < 0.05. The number of wins required for a comparison of *k* = 25 experimental results are [32]: 18 wins for (the significance level) *α* = 0.05 (i.e., 95% confidence level) and 17 wins for a less stringent *α* = 0.1 (i.e., 90% confidence level), respectively. In addition, for a more stringent setting of *α* = 0.01 (i.e., 99% confidence interval), a total of 19 wins is required.

### 4.2 Benchmark data

A total of 10 data sets are used in the experiments. The details of each data set, i.e., B1 to B10, are shown in Table 4, including the number of instances and features as well as the imbalanced ratio (IR) information.

**Table 4:** List and descriptions of benchmark datasets.

Data set	Ref	Problem	Instances	Features	IR
B1	[29]	abalone-17_vs_7-8-9-10	2,338	8	39.3
B2	[29]	abalone-20_vs_8-9-10	1,916	8	72.7
B3	[29]	flare-F	1,066	11	23.8
B4	[28]	pima	768	8	1.9
B5	[29]	ring	7,400	20	1.0
B6	[28]	spambase	4,597	57	1.5
B7	[29]	twonorm	7,400	20	1.0
B8	[29]	winequality-red-4	1,599	11	29.2
B9	[29]	winequality-white-3-9_vs_5	1,482	11	58.3
B10	[30]	Credit card transactions by European cardholders	284,807	30	577.9

The accuracy rates and F1 scores are shown in Tables 5 and 6, respectively. In general, the BKS results are slightly higher than those from majority voting for both performance indicators.

**Table 5:** Accuracy rates.

Data set	BKS	Voting
B1	0.9734	0.9717
B2	0.9864	0.9864
B3	0.9453	0.9439
B4	0.7204	0.7205
B5	0.9541	0.9466
B6	0.9514	0.9498
B7	0.9782	0.9352
B8	0.9501	0.9521
B9	0.9823	0.9809
B10	0.9981	0.9980

**Table 6:** F1 scores.

Data set	BKS	Voting
B1	0.9863	0.9855
B2	0.9931	0.9931
B3	0.9715	0.9704
B4	0.7675	0.7700
B5	0.9539	0.9451
B6	0.9601	0.9588
B7	0.9782	0.9353
B8	0.9742	0.9753
B9	0.9911	0.9904
B10	0.9991	0.9990

**Fig 3:**
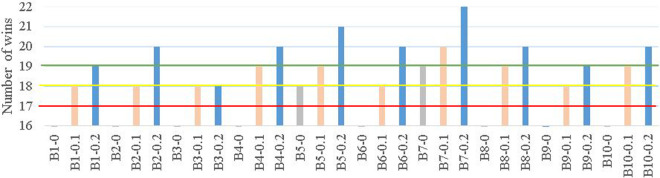
Number of BKS wins over majority voting in data sets with and without noise (red, yellow, and green lines indicate the threshold of wins requires for significance level of *α* = 0.1, 0.05 and 0.01, respectively).

To evaluate the robustness of BKS, the data samples are corrupted with noise at 10% and 20% levels. A total of 25 runs are conducted for each data set, and the average results are listed in Table 7. Fig 3 indicates the numbers of wins pertaining to the BKS against majority voting. The three bars for each dataset represent the data with no noise (-0), with 10% noise (-0.1), and with 20% noise (-0.2).

**Table 7:** Accuracy rates with and without noise.

Data set	Noise	BKS	Voting
B1	0%	0.9734	0.9717
	10%	0.9402	0.9263
	20%	0.912	0.8923
B2	0%	0.9864	0.9864
	10%	0.9716	0.9625
	20%	0.9438	0.9296
B3	0%	0.9453	0.9439
	10%	0.9405	0.9221
	20%	0.9334	0.9027
B4	0%	0.7204	0.7205
	10%	0.7391	0.6859
	20%	0.7235	0.6822
B5	0%	0.9541	0.9466
	10%	0.9534	0.9483
	20%	0.9528	0.9460
B6	0%	0.9514	0.9498
	10%	0.8697	0.7874
	20%	0.8627	0.8150
B7	0%	0.9782	0.9352
	10%	0.9468	0.9226
	20%	0.9365	0.9004
B8	0%	0.9501	0.9521
	10%	0.9155	0.8967
	20%	0.8650	0.8236
B9	0%	0.9823	0.9809
	10%	0.9469	0.9341
	20%	0.8910	0.8709
B10	0%	0.9981	0.9980
	10%	0.9629	0.9535
	20%	0.9571	0.9230

To evaluate whether BKS performs better than majority voting from the statistical perspective, a two-tailed sign test is used, as detailed in Section 4.1. Fig 3 shows the number of wins of BKS over majority voting from the experimental results (plotted at 16 wins and above). BKS achieves at least 18 wins out of 25 experimental runs in all ten noisy data sets (10% and 20% noise levels), indicating its superior performance over majority voting in undertaking noisy data samples for α = 0.05 (95% confidence level). When a more stringent statistical significance level of α = 0.01 (i.e., 99% confidence level) is used for evaluation, BKS outperforms majority voting in 9 out of 10 data sets with a noise level of 20%. This outcome positively indicates the usefulness of BKS over majority voting in mitigating the negative effect of noise in performance.

To ascertain the effectiveness of BKS with other methods in the literature, a comparison of the F1 score with the published results of GEP [26] and CUSBoost [33] is shown in Table 8. CUSBoost [33] achieves the worst performance, while GEP [26] achieves close results as compared with those from BKS and majority voting. Overall, BKS achieves the highest F1 scores in four out of six data sets, while the scores of the remaining two are a little lower by 0.01 as compared with those of majority voting.

**Table 8:** Comparison of F1 scores with literature (best in bold).

Dataset	BKS	Voting	GEP [26]	CUSBoost [33]
B1	**0.9863**	0.9855	0.9048	0.3231
B2	**0.9931**	0.9931	-	0.3363
B3	**0.9715**	0.9704	0.927	0.1809
B4	0.7675	**0.7700**	-	0.5543
B8	0.9742	**0.9753**	0.9005	0.0939
B9	**0.9911**	0.9904	0.8964	0.1674

### 4.3 Real-world data

This evaluation focuses on real financial transaction records (available in [34]) from September to November 2017 in a Southeast Asia financial firm. As indicated in [35], Southeast Asia is one of the fastest growing regions over the years, with a gross domestic product growth rate of over 6%. In this experiment, a total of 60,595 transaction records from 9,685 customers are available for evaluation. The transactions cover activities in 23 countries, with various spending items ranging from online website purchases to grocery shopping. A total of 28 transactions have been identified by the firm and labeled as fraud cases, with the remaining being genuine, or non-fraud cases.

Each transaction record consists of the account number, transaction amount, date, time, device type used, merchant category code (MCC), country, and type of transaction. The account number is anonymized to ensure privacy of customers. In addition to the nine original features, feature aggregation is conducted to generate eight new features. These aggregated features utilise the transaction amount, acquiring country, MCC, and device type over a period of three months. A summary of the features is shown in Table 9.

**Table 9:** List of features and description.

No	Features	Description
1	Account Number	Anonymized account number
2	Transaction Amount	Amount spent in the transaction
3	Transaction Date	Date of said transaction
4	Transaction Time	Time of said transaction
5	Device Type	Type of device used for transaction
6	MCC	Merchant category code
7	Acquiring Country	Country where transaction took place
8	For Country	Country where card was issued
9	Transaction Type	Sale or cancellation
10	Transaction Amount Count	Count of transactions by cardholder
11	Transaction Amount Sum	Sum of total transactions by cardholder
12	Acquiring Country Count	Count of unique acquiring country
13	Acquiring Country Sum	Sum of acquiring country for transaction
14	MCC Count	Count of all MCC
15	MCC Sum	Sum of specific MCC for transaction
16	Device Type Count	Count of different device types used
17	Device Type Sum	Sum of specific device type used for transaction

**Fig 4:**
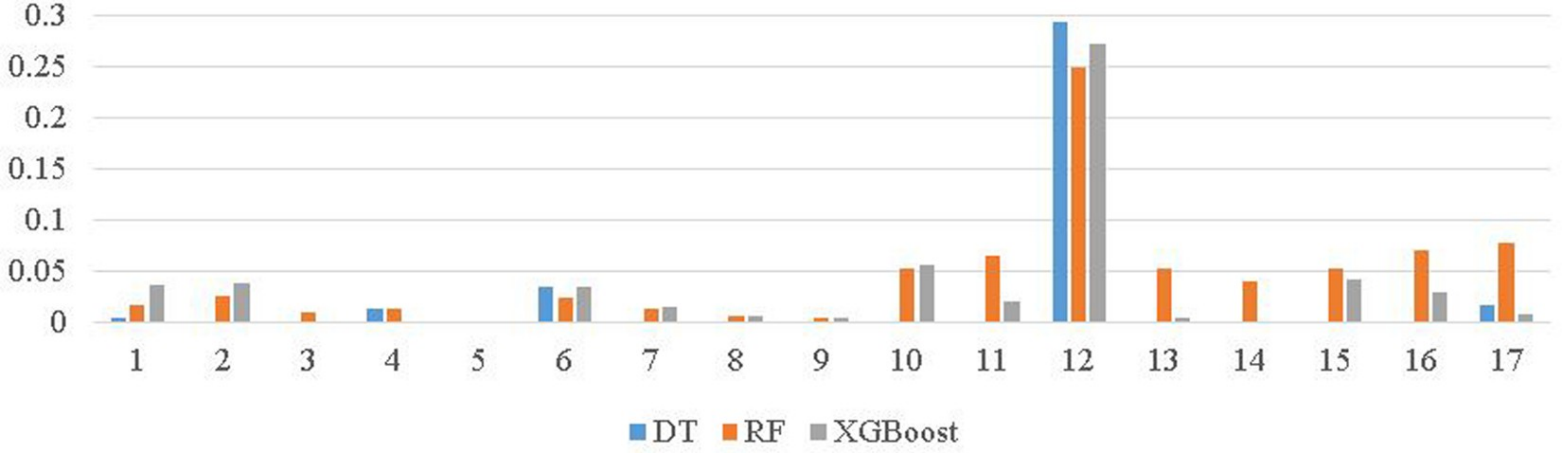
Feature importance using DT, RF, and XGBoost.

Feature importance scores can provide useful information of the data set. The scores can highlight the relevance of each feature for classification. Based on the 17 features, we carry out a feature importance study using the Decision Tree (DT), Random Forest (RF), and XGBoost classifiers. Fig 4 illustrates the results. It can be observed that all the features depict different levels of importance, and feature 12 (i.e., the count of unique acquiring country) appears to be the most important feature in all three classifiers. The remaining aggregated features (features 10 to 17) generally have slightly higher importance scores as compared with those of the original features.

Similar to the benchmark data experiment, noise is added with increment of 10% to 40% to this real-world data set. Table 10 summarizes the results. BKS outperforms majority voting when the level of noise increases, indicating its robustness against noisy data. When the noise level increases to 20% and above, BKS outperforms majority voting 18 times (20% and 30% noise) and 19 times (40% noise), respectively. This outcome positively signifies the statistical superior performance of BKS over majority voting at 95% confidence level (*α* = 0.05) for undertaking noisy data (20% noise and above) in this real-world experiment.

**Table 10:** Accuracy rates and BKS wins with noise added.

Noise	BKS	Voting	BKS Wins
0%	0.9993	0.9993	0
10%	0.9961	0.9907	9
20%	0.9872	0.9771	**18**
30%	0.9699	0.9576	**18**
40%	0.9656	0.9511	**19**

Table 11 lists that F1 scores of the experiments. When no noise is added, the F1 scores for both BKS and voting are the same. Again, for noisy data sets, BKS consistently achieves higher F1 scores, as compared with those from majority voting.

**Table 11:** F1 scores with noise added.

Noise	BKS	Voting
0%	0.9996	0.9996
10%	0.9970	0.9963
20%	0.9935	0.9881
30%	0.9845	0.9779
40%	0.9822	0.9762

In addition to the experiments with additive noise, two experiments with under-sampling methods are conducted. Two different ratios of minority (fraud transactions) to majority (genuine transactions) are evaluated, i.e., 1:100 and 1:500, and the overall results are shown in Table 12. Obviously, under-sampling does not help improve the voting results, while the use of 1:100 ratio enhances the BKS results slightly, as the data set is much more balanced, as compared to the original ratio.

**Table 12:** Accuracy rates with different ratios of minority to majority samples.

Sampling	BKS	Voting
Original	0.9993	0.9993
1:100	0.9995	0.9981
1:500	0.9993	0.9980

## 5. Conclusions

A multi-classifier system has been designed to address the classification challenge pertaining to credit card fraud. Specifically, the combination of a hierarchical agent-based framework with the BKS as a decision-making method has been constructed for classifying transaction records of credit cards into fraudulent and non-fraudulent cases. This combination allows the accumulation of knowledge and yields better results over time. To evaluate the proposed multi-classifier system, a series of experiments using publicly available data sets and real financial records have been conducted. The results from the ten benchmark data sets indicate the performance of BKS is better than that of the majority voting method for decision combination. In addition to noise-free data, noise up to 20% has been added to the data samples, in order to evaluate the robustness of the proposed method in noisy environments. Based on the statistical sign test, the BKS-based framework offers statistically superior performance over the majority voting method.

For the real transaction records from a financial firm, up to 40% noise has been added to the data samples. When the noise levels reach 20% and above, the BKS-based framework outperforms the majority voting method, with statistical significance at the 95% confidence level, as ascertained by the sign test. Based on the outcomes from both benchmark and real-world data, the proposed BKS-based framework is effective for detecting fraudulent credit card cases.

In future work, we will address several limitations of the current BKS models. Firstly, it is possible for the BKS table to contain empty cells, leading to no prediction for a given data sample. This observation generally occurs when the number of classifiers increases, i.e., a larger knowledge space is formed. In addition, noisy data sets, particularly noise in class labels, result in inaccurate information captured in the BKS cells, leading to erroneous predictions. We intend to exploit probabilistic methods, such as Bayesian inference, to interpret the BKS prediction and enhance its robustness in undertaking noisy data classification problems.

Additionally, we will investigate imbalanced data issues using a combination of over-sampling and under-sampling techniques. The effect of these different techniques toward classification performance will be analyzed and compared systematically using statistical hypothesis tests. We will also develop an online version of the proposed model. The model will be able to learn data samples on-the-fly and keep improving its prediction accuracy incrementally. This online learning model will be applied to various financial problems as well as other classification tasks.
